# Organoids in parasitology: a game-changer for studying host–nematode interactions

**DOI:** 10.1017/S0031182025100620

**Published:** 2025-09

**Authors:** Matias Gaston Perez, Diana Coman, Joana Neves, Collette Britton

**Affiliations:** 1 School of Biodiversity, One Health and Veterinary Medicine, College of Medical, Veterinary and Life Sciences, University of Glasgow, Glasgow, UK; 2 Centre for Host–Microbiome Interactions, Faculty of Dentistry, Oral & Craniofacial Sciences, King’s College London, London, UK

**Keywords:** gastrointestinal nematodes, host–parasite interactions, immune modulation, intestinal epithelium, *in vitro* models, organoids, stem cells

## Abstract

Gastrointestinal (GI) nematode infections represent a significant health burden globally, affecting both humans and livestock. Traditional *in vitro* models to study host–parasite interactions, such as immortalized cell lines, have limitations that hinder the full understanding of these complex relationships. Organoid technology has emerged as a promising alternative, offering a physiologically relevant platform to study host–nematode interactions *in vitro*. Organoids are three-dimensional structures comprising differentiated cell types that recapitulate features of the corresponding organ. Technological advances for growing, maintaining and manipulating organoids have increased their applications to model infections, inflammation and cancer. This review discusses recent work using GI organoids to advance understanding of nematode–host interactions and modulation of GI epithelial cells. Additionally, we review studies that co-cultured GI organoids with innate lymphoid cells to study epithelial-immune cell cross-talk in the context of nematode infection. By bridging the gap between reductionist cell culture systems and whole-organism studies, organoids offer a powerful platform for investigating complex host–nematode interactions, and for developing and screening novel therapeutics.

## Graphical abstract

**Figure:**
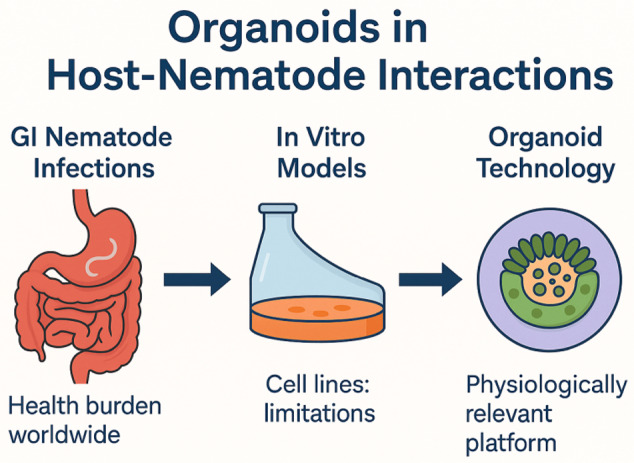

## Introduction

Gastrointestinal (GI) nematode infections pose a major health burden, affecting over 1.5 billion people, particularly in tropical and subtropical regions with inadequate sanitation (World Health Organization 2024). These macroparasites have evolved intricate life cycles that enable them to successfully colonize the GI tract of humans and other mammals. Prominent human species such as *Ascaris lumbricoides, Trichuris trichiura* and *Necator americanus* contribute to malnutrition, stunted growth and cognitive impairments, especially in children (Servián et al. 2024). Similarly, in livestock, GI nematodes cause significant economic losses due to reduced weight gain, decreased milk production and increased mortality (Strydom et al. 2023; Vande Velde et al. 2018). For example, *Haemonchus contortus* and *Ostertagia ostertagi*, which infect sheep and cattle, respectively, result in billions of dollars in losses annually (Charlier et al. 2020). Additionally, in horses, nematodes such as *Strongylus vulgaris* and Cyathostominae cause severe colic and life-threatening complications (Corning 2009).

GI nematodes are specially adapted to evade host defences, migrate through tissues and establish a niche, often leading to chronic infections (maizels And Gause 2023). Given the increasing resistance of GI nematodes to anthelmintic drugs, understanding how these parasites establish infection and evade host immunity is important for developing more sustainable control strategies in both human and veterinary medicine (Charlieret al. 2022; Hotez and Herricks 2015; Nielsen 2022; Vegvari et al. 2021). The intestinal epithelium plays a central role in host–parasite interactions by detecting pathogens and orchestrating protective mechanisms, leading to increased mucus secretion and enhanced peristalsis – collectively known as the ‘weep and sweep’ response (Bąska and Norbury 2022). Several studies in recent years identified epithelial tuft cells, a type of chemosensory secretory cell, as initial ‘sensors’ of parasite infection in the small intestine (SI) (Gerbe et al. 2016; Howitt et al. 2016; von Moltke et al. 2016). Further understanding of GI epithelial cell responses to nematode infection, and their interaction with immune cells is important in gaining insight into the initiation of host immune responses required for effective parasite control.

Mucosal tissues of the GI tract initiate innate and adaptive type 2 immune responses to nematode infection (Artis and Grencis 2008). Alarmins interleukin (IL)-25 from GI tuft cells, thymic stromal lymphopoietin (TSLP) from epithelial cells, and IL-33 from epithelial, endothelial and inflammatory immune cells, activate Group 2 innate lymphoid cells (ILC2s) (Hardman et al. 2013). Once activated, ILC2s play a crucial role as an early source of type-2 cytokines, in particular IL-4 and IL-13 which act in a feed forward loop to increase the numbers of tuft and mucous-secreting cells in the GI epithelium (Fallon et al. 2006; Molofsky and Locksley 2023). Cytokines from ILC2 cells also act to recruit effector cells, including eosinophils, alternatively activated macrophages, and IgE- and IgG1-producing B cells, to resolve the infection, as well as promote tissue repair (Hartung and Julia 2022; Vivier et al. 2018). In the face of these protective mechanisms, nematodes have evolved multiple strategies to manipulate or suppress host immunity, enabling them to persist within their host for extended periods (Maizels et al. 2018). Much of our knowledge of nematode immunomodulation stems from studying worm excretory–secretory (ES) products and their effects on mammalian cells using immortalized cell lines or *in vivo* animal models (Maizels et al. 2018). Organoids (‘mini-tissues’) provide a new and powerful *in vitro* platform for studying host–pathogen interactions and immunomodulation. This review focuses on the application of organoids to advance understanding of host–parasite interactions and immunomodulation in the GI tract.

## The need for more physiological model systems

Traditional cell culture models are widely used due to their ease of maintenance and scalability; however, they have significant limitations. Cell lines are often derived from cancerous or immortalized tissues, which compromise their ability to accurately mimic native epithelial cells (AGUILAR et al. 2021). For example, Caco-2 and HT-29 cell lines are derived from human colorectal adenocarcinoma and are used as *in vitro* models of gut epithelium (Haddad et al. 2023). However, they lack proper differentiation and fail to replicate the structural and functional complexity needed for studying host–parasite interactions. Even when grown in three-dimensional (3D) cultures, these cell lines do not capture the cellular diversity of the intestinal epithelium, limiting their utility in modelling *in vivo* conditions.

Primary cell cultures and tissue explants are physiologically relevant alternatives, as they maintain native tissue architecture and functionality (Schlaermann et al. 2016). However, their limited expansion and requirement to be derived from fresh tissue present significant challenges, limiting their application in long-term studies. Animal models, particularly rodents, have been instrumental in providing valuable insights into both local and systemic host responses to parasitic infections of humans and animals (Mukherjee et al. 2022). For example, the murine whipworm *Trichuris muris* is a useful model for the human parasite *Trichuris trichiura*, allowing researchers to study host immunity, parasite biology and host genetics in a controlled setting (Klementowicz et al. 2012). However, for many parasitic helminth species, no appropriate model system supports parasite development and maturation that mimics *in vivo* infection. Additionally, animal use comes with significant challenges, including the need for specialized housing, high maintenance costs, ethical concerns, and limited ability to track host–parasite interactions over the course of infection (de Graeff et al. 2019).

Organoid-based models have the potential to overcome these limitations by providing a controllable and scalable platform to investigate host–parasite interactions and, for some parasites, initial invasion. The development of organoids from tissue stem cells of human, murine, ruminant and other animal species presents a promising alternative, offering a physiologically relevant system for studying important helminth infections, while reducing reliance on murine models. There is increasing interest in organoid-based systems as alternative models, to bridge the gap between cell culture approaches and whole-organism studies.

## Organoid types

Organoids are self-organising 3D structures developed from tissue stem cells or induced pluripotent stem cells. Using culture media containing defined growth factors and nutrients, the stem cells differentiate and form 3D structures with similar organization and function to the tissue of origin (Lancaster and Knoblich 2014; Sato and Clevers 2015; Sato et al. 2009). Organoids have been grown from a range of tissue stem cells including gut (abomasum in ruminants), SI, colon, lung, liver, kidney, brain, bile duct, retinal and pancreas (Kawasaki et al. 2022; Tang et al. 2022). They can be propagated and passaged as long as stem cells are present and can be cryopreserved, enabling the establishment of organoid biobanks (Perrone and Zilbauer 2021).

**Figure 1.**
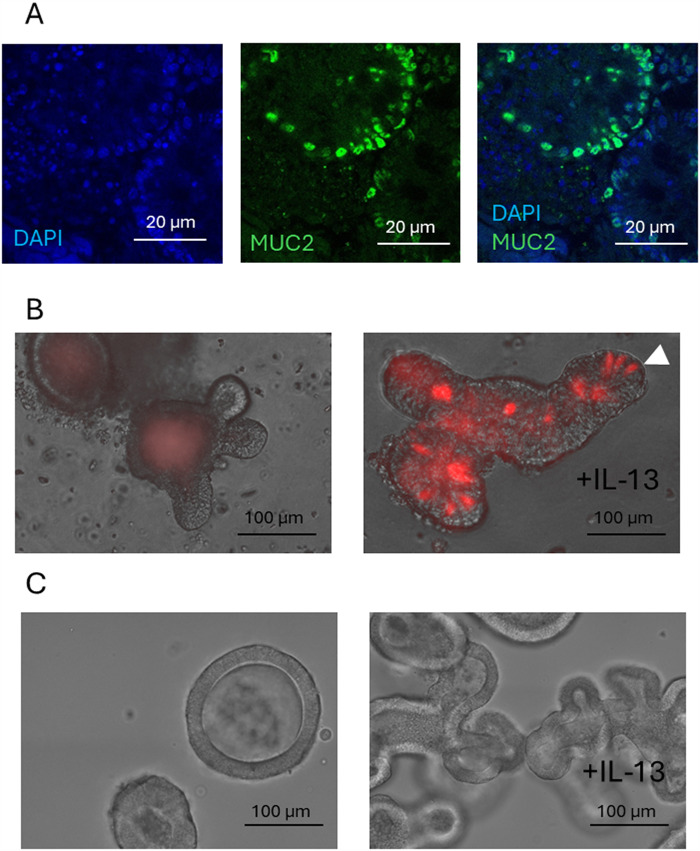
Differentiation of gastrointestinal organoids and cell type identification. (A) Confocal microscopy image showing MUC2⁺ cells in ovine small intestinal (SI) organoids cultured in organoid growth medium (OGM) for 4 days. (B) Representative images of *Dclk1*-tdTomato⁺ tuft cells (red tdTomato^+^ cells indicated by arrowhead) in murine SI organoids, either untreated or treated with IL-13 in OGM for 4 days. (C) Representative image of ovine abomasal (gastric) organoids cultured in OGM for 4 days, with or without IL-13 treatment.

Importantly, although organoids typically grow as 3D structures embedded in matrices such as Matrigel, they can also be dissociated into single cells or small clusters and cultured as two-dimensional (2D) monolayers. This process involves plating organoid-derived cells onto coated culture plates, where they attach and form confluent monolayers. While these monolayers do not replicate the full 3D architecture of the tissue, they retain many aspects of cellular differentiation and polarization (Duque-Correa et al. 2022; White et al. 2024) and offer the advantage of more direct access to the apical (luminal) surface (Wang et al. 2017). For studying the GI tract, organoids have been developed using stem cells derived from gastric (abomasal), duodenal, ileum, jejunum, caecum and colon tissue (Barker et al. 2010; Jung et al. 2011; Smith et al. 2021; Williamson et al. 2013; Yui et al. 2012), with subsequent differentiation into secretory epithelial cell types (Paneth, tuft, goblet (mucous producing) and enteroendocrine cells), and absorptive enterocytes (Fukuda et al. 2014; Sato and Clevers 2015). The different cell types can be identified by immunohistochemistry on fixed organoids using antibodies to specific marker proteins, such as anti-POU2F3 or anti-DCLK-1 for tuft cells, anti-MUC2 for mucous cells and anti-LGR5 for stem cells (Gerbe et al. 2016; Perez et al. 2025). Additionally, reporter organoids expressing a fluorescence gene under the control of a specific cell marker gene promoter enable analysis and imaging in real-time. Examples of 3D GI organoids stained with antibodies or expressing a reporter gene (tuft cell *Dclk-1* promoter driving tdTomato) are shown in Figure 1, and detailed in Perez et al. (2025).

## Considerations for studying nematode–host interactions using organoids

Developing organoid models to study parasites requires consideration of fundamental aspects of host–parasite interactions: what is the natural site of infection within the host, which host cell types are primarily targeted and/or modulated by the parasite, are there differences between hosts, which parasite life-stage(s) or molecular components are most relevant for investigation, how can parasites or parasite molecules be delivered into organoids and how can parasite-mediated effects be assayed? We will firstly consider host tissue and cell types and in the next section discuss organoid manipulation and monitoring of parasite-mediated effects on organoids.

### Infection site tissue type

GI nematodes occupy various niches within the host, including lumen or submucosa depending on the parasite developmental stage and species, where they can induce substantial alterations in epithelial cells. While adult worms are confined to the intestinal lumen, infective third-stage larvae (L3) of some nematode species actively penetrate the GI epithelial barrier, as seen with *Heligmosomoides polygyrus, T. trichiura* and *Strongyloides stercoralis*, which invade the submucosa. Blood-feeding nematodes, such as *H. contortus* and human hookworms *Ancylostoma duodenale* and *N. americanus*, attach to the intestinal mucosa causing mechanical damage and blood loss. An intriguing aspect of some GI nematodes, such as *Ascaris* and hookworms, is the requirement for hepato-tracheal migration of L3 prior to maturation in the SI (Read and Skorping 1995). These diverse interactions highlight the range of host–parasite relationships that influence disease pathogenesis and immune responses, and the relevance of developing organoids from the appropriate host tissue.

When studying parasite effects on host organoids, it is important to recognize that nematodes exhibit specific tropisms for distinct regions of the GI tract, certain cell types, and may remain in the lumen or invade submucosal tissue. A recent study by White et al. (2024) established confluent 2D SI monolayers to investigate interactions between *Heligmosomoides bakeri* L4 or adult stages and host duodenal cells. Notably, when the basal side of the epithelial cells was exposed to L4 or adult *Heligmosomoides bakeri*, the cellular response was greater than when the apical side was exposed, perhaps reflecting differences in expression of Pattern Recognition Receptors between the apical and basal surfaces (Price et al. 2018). Additionally, L4 parasites, which develop submucosally, induced greater upregulation of epithelial interferon-stimulated genes and repair genes compared to adult worms, which reside in the lumen. These findings underscore the importance of considering the tissue site of infection, orientation of epithelial cells and the relevant nematode developmental stages to dissect the dynamics of infection at both the host and parasite levels.

### Parasite immunomodulation in organoids

While organoids mimic features of the tissue of origin, they lack the complexity of associated mesenchymal, stromal or immune cells. Parasite–epithelial–immune cell interactions can be examined using co-culture models (see below) or, alternatively, specific cytokines can be added to organoid cultures. For example, addition of type-2 cytokines IL-4 and IL-13 to murine SI organoids promotes organoid differentiation (budding), specifically expansion of secretory tuft and mucous cell types (see Figure 1B, C and Drurey et al. 2021; Kato-Atar et al. 2022). This mimics the *in vivo* effects of these cytokines on epithelial cells during nematode infection and provides a useful platform to examine infection-associated changes and parasite-mediated immunomodulation. Using this approach, Drurey et al. (2021) and Karo-Atar et al. (2022) demonstrated that *H. polygyrus* adult ES products suppress IL-4/IL-13-induced tuft and goblet cell expansion, demonstrating the parasite’s ability to dampen host innate responses by directly acting on epithelial cells. In contrast to IL-4/IL-13 treatment, GI organoids treated with IL-22, which is mainly produced by T helper 1 (Th1), Th17 and group 3 innate lymphoid cells (ILC3) (Keir et al. 2020; Perusina Lanfranca et al. 2016) results in reduced organoid budding, a dark appearance and increased expression of goblet cell marker Resistin-like beta molecule (RELM-β) (Lindholm et al. 2022). Interestingly, Lindholm et al. (2022) further demonstrated that BMP signalling within intestinal organoids acts as a feedback mechanism to limit IL-13-induced tuft cell hyperplasia, highlighting how organoids can effectively model cytokine-driven epithelial differentiation and regulatory pathways observed *in vivo*, providing a valid platform to study immunomodulation.

### Organoids to study the establishment of infection

*In vitro* culture of parasitic helminths remains a challenge, making it difficult to examine helminth development and tissue invasion. Elegant studies from the Duque-Correa group (Duque-Correa et al. 2022) established 2D caecal epithelium grown in transwells to study nematode larval invasion and development. They showed that *T. muris* L1 larvae could degrade secreted mucus and invade intestinal epithelial cells within murine caecal organoids (caecaloids), replicating the early infection processes that occur *in vivo* and establishing the first *in vitro* system for whipworm infection. This was achieved using 2D epithelial cell cultures, derived from 3D organoids and later grown in transwells, where the apical intestinal surface was accessible to the larvae. The 2D system overcame the challenge of trying to deliver larvae to the lumen (apical side) of 3D organoids, which is prohibited by the relatively large size of most nematode larvae. Further studies using a transgel GI organoid system – grown on hydrogel to generate bilaterally accessible organoids – enabled live observation of epithelial cell infection by *T. muris* L1, accompanied by formation of syncytial tunnels as occurs *in vivo* and subsequent epithelial cell apoptosis (Hofer et al. 2024). This demonstrates the utility and power of organoids to examine in detail the processes of parasite infection and establishment *in vitro*.

Organoid technology can also be applied to other types of helminths; recently, spheroids, 3D structures comprising a single cell type rather than differentiated cell types, were developed to study growth of liver fluke *Fasciola hepatica* newly excysted juveniles (NEJ) (Vitkauskaite et al. 2025). The spheroid culture was grown from HepG2 liver cells and was able to support growth of *F. hepatica*, with development of gut, muscle and tegumental structures, as well as secretion of digestive proteases, similar to fluke development *in vivo*. The NEJ grazed on the peripheral cells, rather than invading the spheroid structure.

### Host differences

Organoids are generated from single stem cells from specific individuals and retain characteristics of the original tissue, such as gene expression profiles and treatment responses (Bresnahan et al. 2020). To ensure robust and reproducible results, it is recommended that experiments include organoid lines derived from at least three biological replicate tissue samples. This practice helps to account for natural biological variability and enhance the reliability of the findings. On the other hand, the characteristics maintained in organoids provide a valuable platform for determining differences between individuals or animal breeds/strains, helping to determine why they respond differently to infections (Grencis 2015). This has application in determining mechanisms of resistance or susceptibility, relevant to animal selective breeding programmes, vaccine development and personalized medicine (Bose et al. 2021).

## Delivery into organoids

While 3D organoids provide a more physiological system than cell culture or 2D monolayers, their 3D structure can make the delivery and uptake of parasites or their products to the apical (luminal) side more challenging. Different approaches have been tested to achieve this and, in some studies, where long-term culture is not a requirement, 2D monolayers are appropriate. In this section, we discuss some of the delivery approaches used, which are summarized in Table 1.

**Table 1.** Delivery approaches used to study interaction of GI nematodes with epithelial cells

Delivery method	Parasite stage or component	Organoid type	References
Microinjection	*T. muris* (adult) EVs	Murine colonic 3D organoids	Eichenberger et al. 2018a; Duque-Correa et al. 2020
	*N. brasiliensis* (adult) EVs	Murine SI 3D organoids	Eichenberger et al. 2018b
Direct addition of nematodes	*T. circumcincta* (L3)	Ovine SI 3D organoids	Smith et al. 2021
	*O. ostertagi* (L3)	Bovine gastric 3D organoids	Faber et al. 2022
	*T. muris* (L1)	Murine SI 2D culture	Duque-Correa et al., 2022
	*T. spiralis* (L1)	Porcine SI 3D organoids	Liu et al. 2024
	*H. bakeri* (L4 and adult)	Murine SI 2D cultures	White et al. 2024
	*P. univalens*, Cyathostominae and *S. vulgaris* (L3)	Equine SI 2D cultures	Hellman et al. 2024
Direct addition of nematode-secreted products	*H. polygyrus* (adult) ES	Murine SI 3D organoids	Drurey et al. 2021; Karo-Atar et al. 2022
	*H. contortus* (adult) ES	Ovine gastric 3D organoids	Perez et al. 2025
		Ovine SI 3D organoids	
		Murine SI 3D organoids	
	*T. spiralis* (L1) ES	Porcine SI 3D organoids	Liu et al. 2024
	*Anisakis* (L3) EVs	Human 2D colonic organoids	Bellini et al. 2024
	MicroRNA − 5352 mimic	Ovine gastric 3D organoids	Perez et al. 2025
		Ovine SI 3D organoids	
		Murine SI 3D organoids	

EVs, extracellular vesicles; ES, excretory–secretory products.

### Microinjection

Microinjection was one of the pioneering techniques applied to organoids, allowing the introduction of molecules directly to the apical side of 3D organoids. The first studies to focus on host–parasite interactions used microinjection to deliver extracellular vesicles (EVs) purified from ES products of adult murine whipworm, *T. muris*, into murine colonic organoids (Eichenberger et al. 2018a). Following injection into the organoid lumen, labelled EVs were detected within the cytoplasm of organoid cells. Cellular uptake was observed at 37°C but not at 4°C, indicating this is an active process. Similar findings were observed using microinjection to deliver EVs from *N. brasiliensis* adult ES into murine SI organoids (Eichenberger et al. 2018b). While effects directly on organoids were not reported in these studies, *in vivo* administration of EVs from *N. brasiliensis* ES, but not *T. muris*, protected mice from chemically induced colitis, with reductions in pro-inflammatory cytokines IL-6 and IFN-γ (Eichenberger et al. 2018b). By performing microinjection of EVs from *T. muris* into murine caecaloids followed by RNA sequencing, Duque-Correa et al. (2020) showed that anti-inflammatory effects, including down-regulation of Type I interferon signalling, can be mediated directly on the caecal epithelium in the absence of immune cell types. The specific parasite-derived molecules responsible for these effects are not yet known but may involve proteins and/or small RNAs within EVs.

Microinjection allows precise targeting of specific regions within the organoid; however, it has several limitations. The variability in organoid size can lead to inconsistencies in injection efficiency, making the technique less reliable. Additionally, microinjection is labour-intensive and requires skilled researchers and specialized equipment. It is also impractical to deliver nematodes into the organoid lumen, as the limited luminal volume of organoids is insufficient to accommodate most nematode parasites (Duque-Correa et al. 2020).

### Apical out organoids

An alternative for delivering parasites or ES products to the apical surface is to invert the polarity of 3D organoids using a method developed by Co et al. (2019). This involves removing the extracellular matrix (ECM) from the Matrigel (basement membrane extract), which is added to stem cells to support organoid formation (Co et al. 2021). Organoids are then incubated in low-binding culture plates, which induce a reversal of polarity (apical-out) while preserving barrier integrity and key functional characteristics. This technique was successfully applied to abomasal and SI organoids from different animal species (Smith et al. 2021), allowing access to the apical surface for experimental manipulation. However, the host-driven mechanisms that govern organoid invagination and polarity reversal remain poorly understood and apical-out organoids do not differentiate in the same way as apical-in organoids (Paužuolis et al. 2024). Smith et al. (2021) were able to demonstrate infection of apical-out organoid cells with the bacterial pathogen *Salmonella typhimurium*, but this method has not yet been explored to study nematode infectivity or development.

### Direct addition of nematode larvae or ES products to 3D organoid culture medium

An alternative to microinjection is the direct addition of nematodes or their secreted products to organoid culture medium. Following incubation, larvae or ES can enter into the organoid lumen and/or cells. This strategy was used to introduce infective L3 of the important veterinary nematodes *Teladorsagia circumcincta* and *O. ostertagi* into ovine or bovine abomasal (gastric) organoids, respectively (Faber et al. 2022; Smith et al. 2021). Strikingly, the L3 larvae transversed the Matrigel and organoid membrane, from the basal (outer) to apical (inner) side into the lumen (Faber et al. 2022; Smith et al. 2021). Larvae survived in the lumen for up to two weeks, were active and invaded organoid cells, but did not develop beyond L3 stage (Faber et al. 2022; Smith et al. 2021). This implies that the organoid model may not fully replicate the route of invasion and/or the *in vivo* conditions that support larval development. Future work could explore the feasibility of 2D monolayers, as used for *T. muris* (Duque-Correa et al. 2022), to promote larval development. Nonetheless, 3D organoids can provide valuable insights into the ability of larvae to interact with and cross epithelial barriers. Intriguingly, organoids exposed to *O. ostertagi* L3 or ES products showed a rapid expansion or ‘ballooning’, within 1 hour of treatment, which is thought to result from fluid influx into the lumen (Faber et al. 2022).

The ease of adding soluble substances to organoid culture medium and then monitoring effects has encouraged greater use of this approach. Several studies (Drurey et al. 2021; Karo-Atar et al. 2022; Perez et al. 2025) showed that ES from adult *H. polygyrus* or *H. contortus* can suppress the expansion of tuft and mucous cells driven by IL-4/IL-13, demonstrating a direct effect of ES on epithelial responses. Identifying the molecules and mechanisms involved has the potential to lead to new therapeutics to block nematode-mediated immunosuppression, while conversely, such parasite molecules could be developed as ‘helminth therapy’ to suppress GI inflammatory conditions, such as inflammatory bowel diseaseor allergies (Ryan et al. 2020). *T. spiralis* L1 larvae or ES were also shown to inhibit pro-inflammatory responses, reducing the pathology induced in SI organoids by porcine epidemic diarrhea virus (Liu et al. 2024).

These studies also revealed that ES from adult *H. polygyrus* or *H. contortus* alone in the absence of cytokine treatment had a strong inhibitory effect on SI organoid budding and development. Large spheroid organoids devoid of differentiated cells were observed following 24 h of exposure to ES or L3 larvae, indicating that nematode-secreted molecules induce de-differentiation of organoids and lead to a fetal-like and/or repair state (Drurey et al. 2021; Karo-Atar et al. 2022; Perez et al. 2025). A similar phenotype had previously been observed following growth of organoids derived from SI stem cells from *H. polygyrus*-infected mice, or after IFN-γ treatment of SI organoids, recapitulating the fetal-like granuloma-associated cells occurring *in vivo* during *H. polygyrus* infection (Nusse et al. 2018). Our laboratory found that spheroid formation occurred only with *H. contortus* ES that had been concentrated (through a 3-kDa membrane), but not with unconcentrated ES, indicating a concentration-dependent effect (Perez et al. 2025), analogous to the localized effects *in vivo*. Spheroid formation in organoids was not observed with ES from adult *N. brasiliensis*, which is cleared rapidly from the murine SI (Karo-Atar et al. 2022), suggesting that epithelial de-differentiation may promote nematode survival in the GI tract. This effect is different to the organoid swelling observed with *O. ostertagi* ES or L3 (Faber et al. 2022), which happens rapidly (within 1 h). For both phenotypes, organoids provide an appropriate *in vitro* system to help understand changes induced in epithelial cells during *in vivo* infection.

### Organoid transfection with parasite small RNAs

The above studies demonstrate the utility of organoids in dissecting biological activity of nematode ES products on the GI epithelium. Previous characterization of nematode ES identified proteins with immunomodulatory activity (Hewitson et al. 2009; Maizels et al. 2018); however, little is known about the biological effects of nematode-secreted small RNAs. Small regulatory microRNAs (miRNAs) modulate gene expression post-transcriptionally by binding to the 3’UTR of their target mRNAs, resulting in inhibition of protein translation and transcript degradation (Chekulaeva and Filipowicz 2009). The repertoires of miRNAs expressed and secreted by a variety of nematode species have been described (Britton et al. 2020; Buck et al. 2014). Our laboratory focussed on a single miRNA, miR-5352, which is conserved across GI parasitic nematodes, but not present in tissue-dwelling nematodes (Gu et al. 2017; Perez et al. 2025). This led our group to hypothesize that miR-5352 may be involved in regulating host gene expression in the GI tract, which we tested by transfecting SI organoids with a mimic of miR-5352.

Efficient delivery of the miRNA mimic into the cytoplasm of epithelial cells required transfection reagents with high efficiency, low toxicity and lipid formulations specifically designed for small RNA delivery. Organoids were exposed to miRNA mimic, labelled with Cy3 fluorescent dye, and transfection reagent (DharmaFECT TM) at 37°C for 1 h before being embedded in Matrigel and returned to culture (Perez et al. 2025). This approach significantly increased the number of transfected epithelial cells compared to using naked miRNA mimics, indicated by Cy3 label inside cells. Importantly, this single GI nematode miRNA suppressed differentiation of tuft and mucous cells in the presence of IL-13, similar to the effect of *H. polygyrus* or *H. contortus* complete ES. Kruppel-like transcription factor gene *Klf-4* was identified as a key target of nematode miR-5352, and KLF-4 expression was significantly reduced in miR-5352 transfected organoids (Perez et al. 2025). KLF-4 promotes cell differentiation by inhibiting Wnt signalling, and loss of KLF-4 leads to stem cell maintenance (Zhang et al. 2006), thus identifying mechanistically how nematode ES can modulate gut cell differentiation. Interestingly, nematode miR-5352 shares its seed sequence (key regulatory sequence of miRNAs) with mammalian miR-92a, which also suppresses *Klf-4,* suggesting that GI nematodes have hijacked a host regulatory pathway to suppress innate cell responses and promote nematode survival.

### 2D monolayers to study host–parasite interactions

Development of 2D monolayers, derived from 3D organoids, provides an alternative, accessible apical surface for studying host–parasite interactions and parasite invasion, as demonstrated by the studies of White et al. (2024) using *H. bakeri*, and Duque-Correa et al. (2022) with *T. muris* L1 larvae. Hellman et al. (2024) utilized equine SI 2D cultures (enteroids) stimulated with IL-4 and IL-13 and exposed the apical side to infective larvae of *Parascaris univalens*, Cyathostominae and *Strongylus vulgaris*. Changes in epithelial cell morphology and an increase in MUC2 expression were observed with *P. univalens* L3, indicating a direct effect of larvae on mucous cells. Using human 2D colonic organoids, Bellini et al. (2024) reported that *Anisakis* L3 EVs have tumorigenic and immunomodulatory capabilities. Following organoid treatment with EVs there was a reduction in the receptor for IL-33, potentially dampening type 2 immunity and inflammatory responses.

These findings underscore the utility of different organoid technologies and delivery routes for advancing understanding of GI nematode infections and modulation of epithelial cells. Developments in organoid technology, including co-culture, will continue to improve their relevance to infection biology and as platforms to screen and evaluate new therapeutics.

## Immune cell co-culture

Studying the effects of GI nematode infection on epithelial organoids has advanced our understanding of host–pathogen interactions; however, it is important to recognize that no cellular compartment reacts in isolation *in vivo*. Thus, to better appreciate the implications of GI nematode infection, additional layers of complexity should be incorporated in organoid-parasite models. Mucosal tissue resident immune cells involved in the mediation of type 2 immune responses are a relevant compartment to introduce to the culture systems.

Helminth infection models have been instrumental in the discovery of ILC2s, the innate counterparts of adaptive T helper 2 (Th2) cells (Moro et al. 2010). ILC2s were initially discovered in the adipose tissue of the peritoneal cavity of mice. Upon infection with *N. brasiliensis*, ILC2s produced IL-13 which facilitated goblet cell hyperplasia in the SI and thus helminth expulsion (Moro et al. 2010). Subsequently, ILC2s were also described in the SI tissue where they mediated IL-13–dependent protection against *N. brasiliensis* (Neill et al. 2010; Price et al. 2010) and *T. muris* (Saenz et al. 2010) by inducing the ‘weep and sweep’ response. Since their discovery, the roles of intestinal ILC2s in helminth immunity have been further characterized. *N. brasiliensis* infection in the intestine promoted the activation of ILC2s through the tuft cell-ILC2 axis (Schneider et al. 2018), where tuft cell-derived IL-25 activated ILC2 which in turn led to the production of IL-13. Additionally, IL-33 and TSLP secreted by epithelial cells upon helminth infection further promoted ILC2 activation and induction of a type 2 immune response, necessary for helminth clearance (Hung et al. 2020). Besides the intestine, the protective roles of ILC2s against *N. brasiliensis* larvae extended to the lung (Turner et al. 2013). In the lung, ILC2s use IL-9 in an autocrine manner to enhance their function and support tissue repair during the recovery phase (Turner et al. 2013). Interestingly, helminths were able to interfere with ILC2 activation even when not co-localized. Upon infection with *H. polygyrus*, activation of ILC2s in the lung was hindered due to the release of immunomodulatory ES products, such as *H. polygyrus* Alarmin Release Inhibitor (HpARI) (Osbourn et al. 2017) and *H. polygyrus* Binds Alarmin Receptor and Inhibits (HpBARI) (Vacca et al. 2020).

**Figure 2.**
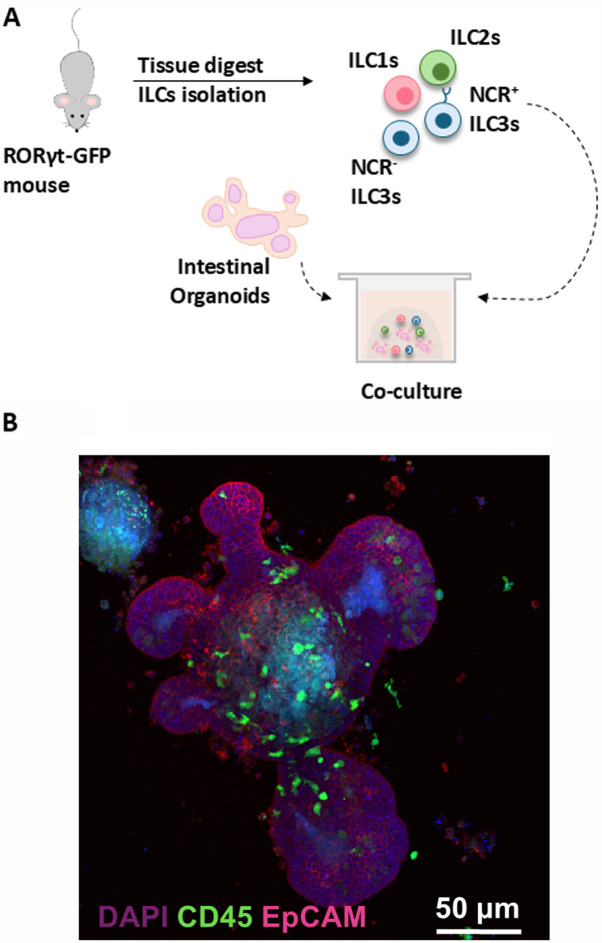
(A) Schematic representation of ILC and organoid co-culture set-up. In brief, small intestinal organoids are generated prior to the co-culture establishment. Organoids are split approximately 48 h prior to placing them together with the ILCs. Pan-ILCs (live, CD45+, Lin- (CD3, CD45R, CD11b, TER-119, Ly-G6, CD19, CD5, CD127+)) or specific ILC groups of interest are isolated from the lamina propria of RORyt – GFP mice, using fluorescence activated cell sorting (FACS). Once isolated, the ILCs are placed together with the organoids, spun down, resuspended in matrigel and placed in a well. Cultures are fed every 2 days until the experimental end point. (B) Confocal microscopy image of ILC precursors, stained for the immune marker CD45, co-cultured with small intestinal organoids, stained for the epithelial cell adhesion molecule (EpCAM) (JOWETT et al.. 2022) (https://creativecommons.org/licenses/by/4.0/#ref-appropriate-credit).

Answering mechanistic questions and dissecting specific immune-epithelial interactions has become streamlined in recent years with the establishment of co-culture models (Kromann et al. 2024). Murine ILC-organoid co-culture models have been generated by isolating ILCs from the lamina propria (Jowett et al. 2021, 2022; Lindemans et al. 2015; Read et al. 2022, 2024) or the mesenteric lymph nodes (Waddell et al. 2019) using florescence activated cell sorting techniques and co-culturing them with GI organoids. Similarly, human models have been established where intestinal ILC2s were co-cultured with intestinal organoids (Möller et al. 2023). Additionally, organoid-maturated human ILC2s have been successfully co-cultured with lung-derived organoids, showcasing the versatility of this model (Jowett et al. 2022). To generate this co-culture, immune cells and epithelial organoids are resuspended together in basement membrane cellular extract to provide the ECM cues which enables the two compartments to interact in a manner similar to that *in vivo* (Figure 2). While this system advanced our understanding of tissue specific immune-epithelial interactions, only looking at the two compartments in a homeostatic setting provides limited applicability and can be enhanced by an additional compartment – parasites.

A tri-culture model involving ILC2s, organoids, and parasites would help elucidate how specific parasites or parasite-derived proteins, miRNAs, or EVs (Buck et al. 2014; Gu et al. 2017; Perez et al. 2025; Soichot et al. 2022) influence gene expression in epithelial and immune cells, and how these changes affect cellular functions, immune responses, and overall host–parasite interactions. Moreover, it would allow us to answer questions related to the functionality and behaviour of ILC2s under helminth infections in a controlled environment. Studies have shown that *Proteobacteria*, a gut microbiome species (Pu et al. 2021), or *N. brasiliensis* (Huang et al. 2018) facilitated the migration of ILC2s from the intestine to the lung. In addition to pathogen-derived signals promoting this migration, tissue-specific imprinting has been implicated in determining the function and fate of post-migratory gut ILC2s in the lung (Jowett et al. 2022). Additionally, under specific microenvironmental conditions, ILC2s displayed a degree of plasticity as they transdifferentiated into ILC1s and ILC3s (Bielecki et al. 2021; Qin et al. 2024). Recent evidence also shows that ILC3s contribute to protective immunity against helminths by promoting tuft cell hyperplasia via RANKL signalling, thereby supporting the ILC2–tuft cell feedback loop (Xu et al. 2025). In future studies, using such a tri-culture model could enable assessment of the impact of parasite and parasite-derived products on epithelial and immune cell modulation and functionality, providing valuable insights into host–parasite dynamics.

Once established and characterized, this tri-culture model could be taken further and additional cellular compartments could be incorporated, such as neurons. Neuroimmune interactions have been previously described in GI nematode infections and ILC2s co-localize with neurons in neuroimmune cell units (Veiga-Fernandes and Artis 2018). Enteric neurons, via their ability to produce neuropeptides such as neuromedin U (Cardoso et al. 2017) and vasoactive intestinal peptide (Nussbaum et al. 2013), promoted the activation of ILC2s, and production of IL-5, IL-9 and IL-13, which in turn led to helminth expulsion. On the other hand, adrenergic neurons produced norepinephrine which inhibited the function of ILC2s, hindering helminth expulsion (Moriyama et al. 2018). ILC2s were reported to synthesise the neurotransmitter acetylcholine in the presence of IL-33 which was required for an appropriate type 2 immune response against *N. brasiliensis* (Chu et al. 2021; Roberts et al. 2021). Interestingly, *N. brasiliensis* and other mucosal-dwelling nematodes synthesize acetylcholinesterases, inhibitors of acetylcholine (Sanderson 1969; Selkirk et al. 2005).

Taken together, future development and application of tri-culture models incorporating epithelial cells, immune cells, and parasites will be essential to advance our understanding of host–parasite dynamics and immune interactions in helminth infections.

## Conclusions and future directions

Organoids provide a powerful platform for studying the complex cellular and molecular responses of the host epithelium to nematode infections and, conversely, how nematodes modulate the host environment. Organoid technology is progressing, and new systems are being developed to increase their physiological relevance and longer-term survival, including the use of air–liquid interface (ALI) models and tissue engineering combined with microfluidics. In ALI models, cells are exposed to air on the apical side while the basolateral side is in contact with culture medium containing differentiation factors. This combination enhances cellular differentiation and 3D formation, as observed in human intestinal organoids which form a defined crypt-villus-like structure under ALI conditions (Baldassi et al. 2021; Ogawa et al. 2025). Tissue engineering strategies, including ECM-embedded scaffolds, bioreactors and organoids-on-a-chip, have been developed as powerful tools for modelling both healthy and diseased organs (Wang et al. 2024) and can support organoid culture and better simulate the natural tissue architecture. Dual-access microfluidic systems facilitate maintenance of nutrients/removal of waste and dead cells, delivery of cytokines to the basal side, and co-culture with additional components such as immune cells, vasculature and microbiome, providing a more physiologically relevant platform (Hofer et al. 2024; Quintard et al. 2024). By maintaining organoids in conditions more akin to *in vivo* conditions, their physiological relevance can be enhanced and their lifespan extended, making them attractive for drug screening and potentially for maintaining parasite lifecycles, a current challenge for most nematode species.

The majority of organoid–nematode studies to date have focused on murine organoid models and model parasite species. Although some studies have begun to use ruminant-derived organoids (Faber et al. 2022; Perez et al. 2025; Smith et al. 2021), there is a clear need to further expand these approaches to include organoids and parasites from a broader range of animal species, as well as humans, to enable true translational impact for both veterinary and human clinical applications.

Nematodes can induce changes in organoid phenotype and cellular composition, which can be detected using antibodies to cell marker proteins (Drurey et al. 2021; Karo-Atar et al. 2022) or use of transgenic reporter organoids (Perez et al. 2025). While the latter require more initial effort to generate, they facilitate the study and imaging of organoid cells in real-time, without the need for fixation or antibodies, which may not be available/applicable for less widely studied host species. Reporter organoids can be generated from genetically modified mice (and potentially other animals) (Perez et al. 2025; Wadosky et al. 2022), or more rapidly using CRISPR-Cas9 gene knock-in technology. This introduces a fluorescent reporter tag to a specific gene of interest by electroporation, and transfected organoids are selected based on fluorescence. Different methodologies have been developed for CRISPR-Cas9 knock-in (Artegiani et al. 2020), with CRISPR-HOT (homology-independent organoid transgenesis) shown to be efficient, enabling real-time cell identification and lineaging. CRISPR-Cas9 or RNA interference mediated by short interfering or short hairpin RNA can also be used to knockdown genes of interest in organoids to examine gene function in a controlled environment. Technologies for organoid culture and co-culture, and CRISPR-Cas9 gene editing, are continuing to expand, providing increasingly sophisticated methodologies to study host cell responses and modulation by parasites and other infectious organisms or disease conditions (Huber et al. 2023; Sun et al. 2021).
